# Expression and Localization of Thrombospondins, Plastin 3, and STIM1 in Different Cartilage Compartments of the Osteoarthritic Varus Knee

**DOI:** 10.3390/ijms22063073

**Published:** 2021-03-17

**Authors:** Daniela Mählich, Anne Glasmacher, Ilka Müller, Johannes Oppermann, David Grevenstein, Peer Eysel, Juliane Heilig, Brunhilde Wirth, Frank Zaucke, Anja Niehoff

**Affiliations:** 1 Institute of Biomechanics and Orthopaedics, German Sport University Cologne, 50933 Cologne, Germany; d.maehlich@dshs-koeln.de; 2 Cologne Center of Musculoskeletal Biomechanics (CCMB), Medical Faculty, University of Cologne, 50931 Cologne, Germany; anne.glasmacher@hotmail.de (A.G.); johannes.oppermann@uk-koeln.de (J.O.); david.grevenstein@uk-koeln.de (D.G.); peer.eysel@uk-koeln.de (P.E.); juliane.heilig@googlemail.com (J.H.); 3 Institute of Human Genetics, Center for Molecular Medicine, Center for Rare Disorders, University of Cologne, Kerpener Str. 34, 50931 Cologne, Germany; ilka.mueller@uk-koeln.de (I.M.); brunhilde.wirth@uk-koeln.de (B.W.); 4 Department for Orthopaedic and Trauma Surgery, Medical Faculty, University of Cologne, 50931 Cologne, Germany; 5 Center for Biochemistry, Medical Faculty, University of Cologne, 50931 Cologne, Germany; 6 Dr. Rolf M. Schwiete Research Unit for Osteoarthritis, Department of Orthopaedics (Friedrichsheim), University Hospital Frankfurt, Goethe University, Marienburgstraße 2, 60528 Frankfurt, Germany; frank.zaucke@kgu.de

**Keywords:** articular cartilage, plastin 3, STIM1, COMP, TSP-1, osteoarthritis

## Abstract

Osteoarthritis (OA) is a multifactorial disease which is characterized by a change in the homeostasis of the extracellular matrix (ECM). The ECM is essential for the function of the articular cartilage and plays an important role in cartilage mechanotransduction. To provide a better understanding of the interaction between the ECM and the actin cytoskeleton, we investigated the localization and expression of the Ca^2+^-dependent proteins cartilage oligomeric matrix protein (COMP), thrombospondin-1 (TSP-1), plastin 3 (PLS3) and stromal interaction molecule 1 (STIM1). We investigated 16 patients who suffered from varus knee OA and performed a topographical analysis of the cartilage from the medial and lateral compartment of the proximal tibial plateau. In a varus knee, OA is more pronounced in the medial compared to the lateral compartment as a result of an overloading due to the malalignment. We detected a location-dependent staining of PLS3 and STIM1 in the articular cartilage tissue. The staining intensity for both proteins correlated with the degree of cartilage degeneration. The staining intensity of TSP-1 was clearly reduced in the cartilage of the more affected medial compartment, an observation that was confirmed in cartilage extracts by immunoblotting. The total amount of COMP was unchanged; however, slight changes were detected in the localization of the protein. Our results provide novel information on alterations in OA cartilage suggesting that Ca^2+^-dependent mechanotransduction between the ECM and the actin cytoskeleton might play an essential role in the pathomechanism of OA.

## 1. Introduction

Osteoarthritis (OA), the most common disease of synovial joints, is characterized by whole joint failure including cartilage degeneration and subchondral bone changes. Multiple risk factors lead to OA. Besides age, gender, genetic factors and metabolic disorders, also excessive mechanical loading caused by malalignment, injuries or obesity and hard physical work or activity are associated with an increased development of the disease [1]. To date, OA is considered to be incurable and current treatment options are insufficient. A better understanding of the molecular pathogenesis of OA is needed to develop novel treatment strategies targeting underlying mechanisms. The most important central pathophysiological process of the disease is a change in the homeostasis of the extracellular matrix (ECM). The ECM is essential for the health and maintenance of cartilage because its integrity and composition accounts not only for the mechanical properties of the tissue but also its function [2]. In addition, the structure and composition of the ECM influences growth factor signaling as well as the metabolic activity and function of chondrocytes, which in turn secrete and maintain the ECM [3]. Besides collagen fibrils and proteoglycan aggregates, the cartilage ECM is mainly composed of glycoproteins that interconnect these suprastructures. Thrombospondins (TSPs) are a family of five oligomeric glycoproteins (TSP-1 to TSP-5) that bind to collagens but are also involved in their secretion and assembly [4,5,6]. They bind Ca^2+^ and undergo calcium-dependent conformational changes that are important for their function. TSPs can have antiangiogenic properties, interact with growth factors and thereby modulate their activity [5]. Finally, chondrocytes can directly attach to TSPs in the ECM via integrin receptors. In early OA, the best-studied family member TSP-5 (also referred to as cartilage oligomeric matrix protein, COMP) is proteolytically degraded but re-expressed at later stages [7]. COMP accelerates collagen fibrillogenesis and regulates chondrocyte proliferation and matrix assembly [4,8]. It is widely used as a serum marker for OA and its level correlates with disease severity, but its function in OA is still unknown [9,10]. TSP-1 is an antiangiogenic factor [11], and OA progression is suppressed through an increased TSP-1 expression via reduction of vascular density in articular cartilage [12].

The tight interaction of the ECM with embedded cells is mediated by the actin cytoskeleton and is of particular importance for cartilage homeostasis. The actin cytoskeleton is a dynamic network of actin and a large number of actin-binding proteins and is required for crucial cellular processes such as the movement of organelles, signal transduction, attachment of the ECM, cellular stiffness and shape, mechanotransduction, endocytosis and cell migration and adhesion [13].

Plastins (PLSs, also known as fimbrins) are a family of actin-binding and -bundling proteins which have an important function in cytoskeleton organization [12,13]. There are three structurally similar and highly conserved plastins that are expressed in different tissues: PLS1 (I-plastin), PLS2 (L-plastin) and PLS3 (T-plastin). PLS1 is expressed in the intestine, colon and kidney, PLS2 in hematopoietic cell lineages and many types of cancer cells, and *PLS3* is ubiquitously expressed in solid tissues [14]. The PLSs are characterized by two actin-binding domains (ABD1 and ABD2) at the C-terminal end, each with two calponin homology units. All plastins bind to F-actin via the ABDs and can cross-link actin filaments into higher-order assemblies such as bundles [15]. The two Ca^2+^-binding EF-hand motifs at the N-terminal end regulate the actin-bundling activity of plastins [16,17]. Overall, PLSs have been shown to play an essential role in cell migration, cell adhesion, endocytosis, mechanotransduction and membrane trafficking [14,15].

PLS3 has been related to bone diseases because mutations in the encoding X-chromosomal gene *PLS3* lead to osteoporosis with a mild to severe phenotype [18,19]. In detail, van Dijk et al. [18] first identified loss-of-function variants in PLS3 as a monogenetic cause of X-linked osteoporosis and osteoporotic fractures in hemizygous males. In heterozygous women, a rare *PLS3* variant was found that resulted in a less pronounced phenotype with milder osteoporosis or normal bone density.

In the last years, various different *PLS3* mutations have been identified to cause osteoporosis with a high variability in disease severity [20,21,22,23,24]. *PLS3* expression has been found in osteocytes [25], osteoclasts [26] and osteoblasts [19]. However, the underlying mechanisms by which mutations in *PLS3* lead to bone alterations are not yet known. A knock-down of *pls3* in zebrafish resulted in malformations in the development of craniofacial bone structures, body axis and tail [18]. In *Pls3* knock-out mice, osteoporosis and decreased bone strength could be detected [26]. The analysis of these animal models revealed that PLS3 might play a role in both bone mineralization by osteoblasts and resorption by osteoclasts. Furthermore, it has been speculated that *PLS3* mutations lead to an altered mechanosensing of osteocytes [18].

Interestingly, about 5% of the general population exhibit increased PLS3 levels in the blood [27]. It has been detected that *PLS3* overexpression is a female-specific protective modifier of spinal muscle atrophy [14,27]. In mice, *PLS3* overexpression resulted in thickening of cortical bone and increased bone strength [26]. It has been shown recently that the Ca^2+^-dependent regulation of actin-bundling by PLS3 is essential for bone formation [16].

There are only very few studies analyzing the role of PLS3 in cartilage. Mäkitie et al. [28] investigated spinal changes in patients with *PLS3* mutations. Although the spinal structures demonstrated severe and progressive alterations in, e.g., vertebra height and shape, pathologies at the intervertebral discs were not present. However, the results were only based on the analysis of the disc area using magnetic resonance imaging. Neugebauer et al. [26] did not report any changes in the spatial organization or longitudinal column alignment of chondrocytes in the growth plate of either 5-day-old *Pls3* knock-out or *PLS3*-overexpressing mice. It should be noted that chondrogenesis or cartilage integrity was not analyzed in more detail in this study. However, Tsolis et al. [29] detected that PLS3 is over-expressed in chondrocytes of knee joint articular cartilage samples from patients with primary OA. Using label-free quantification approach, a 2.5-fold higher abundance of PLS3 was found and validated by immunoblot analysis.

The stromal interaction molecule 1 (STIM1) is a transmembrane protein of the endoplasmic reticulum (ER) that is important for bone homeostasis and maintenance by regulating Ca^2+^ levels in osteoblasts, osteoclasts and osteocytes [30,31] and seems to be involved in chondrogenesis [32,33]. It is a sensor of Ca^2+^ levels in the ER, maintains cellular Ca^2+^ balance and supports Ca^2+^ signaling by initiating the store-operated Ca^2+^ entry process following store depletion [34,35]. The tight regulation of the intracellular calcium concentration is crucial for the actin cytoskeleton, and the fact that STIM-1 has been shown to directly interact with TSP-1 and COMP [36] makes it an attractive candidate that could provide a link between the actin cytoskeleton and the cartilage ECM.

Therefore, the purpose of the present study was to investigate the localization and expression of the Ca^2+^-dependent proteins COMP, TSP-1, PLS3 and STIM-1 in human cartilage samples with different degrees of cartilage degeneration. To do that, we conducted a topographical analysis of the cartilage from the medial and lateral compartment of proximal tibial plateaus from patients with advanced varus knee OA. It has been shown that in a varus knee OA is more pronounced in the medial compartment of the tibial plateau compared to the lateral compartment as a result of an overloading through the malalignment [37,38]. The separate analysis of both compartments should give additional insights into changes that are either dependent on mechanical loading or on the progress of cartilage degeneration.

## 2. Results

### 2.1. OARSI-Score

**Figure 1:**
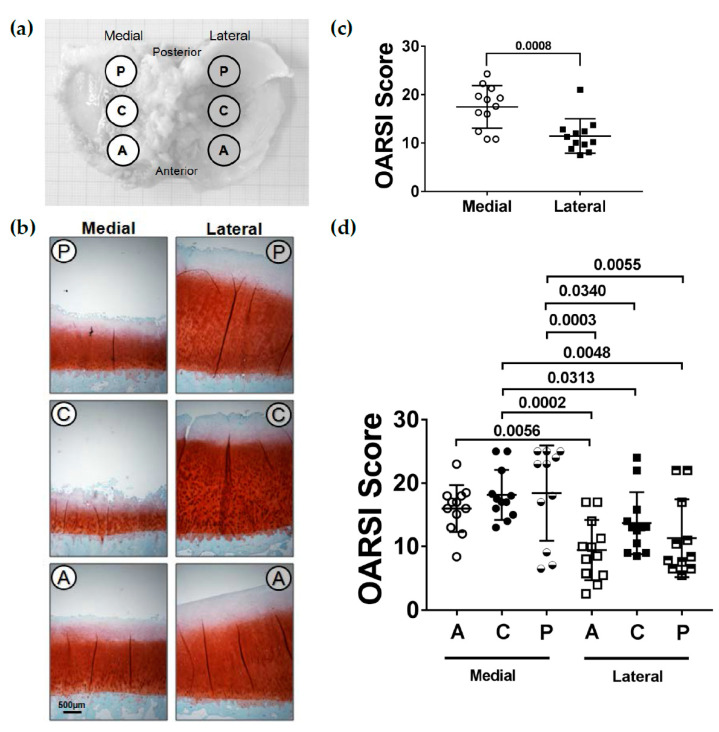
Histopathological Osteoarthritis Research Society International (OARSI) score of the articular cartilage from the medial and lateral tibial compartments. (**a**) Schematic figure depicting the different subregions at which osteochondral plugs were harvested (A: anterior, C: central, P: posterior). (**b**) Representative Safranin O/Fast green-stained sections of the different subregions. (**c**) Total OARSI score determined for the medial and lateral tibial compartments (*n* = 12). (**d**) Total OARSI score determined for the different subregions (A: anterior, C: central, P: posterior) at the medial and lateral tibial compartments (*n* = 11–12 per subregion). Values are represented as scatter plots with indicated mean. Significance between medial and lateral tibial plateaus was evaluated using the paired *t*-test and between subregions using the ANOVA with repeated measurements and Duncan’s multiple range test for post-hoc analysis.

Sixteen patients (8 females and 8 males, 67 ± 9 years) with primary varus knee OA underwent replacement surgery, allowing the collection of the tibial plateaus for the current analysis. Varus knee alignment leads to an unequal loading of the tibial plateau and therefore alterations were always compared between the medial and lateral compartments. In addition, three subregions (A: anterior, C: central, P: posterior) in each compartment were evaluated (Figure 1a,b). To determine the severity of articular cartilage degeneration, the Osteoarthritis Research Society International (OARSI) OA histopathology grading score [39] was applied on Safranin O/Fast green-stained sections of the different subregions of the human tibial plateau. The total OARSI score was significantly (*p* = 0.0008) higher in the medial compared to the lateral compartment (Figure 1c). When the different subregions of the medial and lateral compartments were compared, both the central and the posterior subregions of the medial compartment were significantly (*p* < 0.05) more affected compared to all subregions of the lateral compartment (Figure 1d). The anterior subregion of the medial compartment had a significantly (*p* = 0.0056) higher OARSI score compared to the anterior subregion of the lateral compartment. The OARSI score clearly demonstrated that the medial compartment was more affected by OA than the lateral compartment.

### 2.2. Localization of COMP

**Figure 2:**
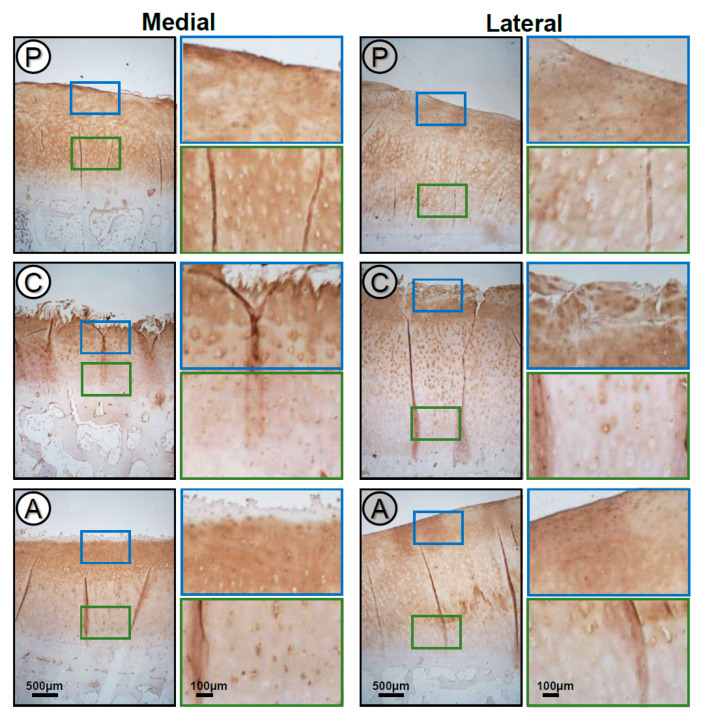
Localization of COMP in the different articular cartilage subregions (A: anterior, C: central, P: posterior) of the medial and lateral tibial compartments. Magnifications of the upper (blue) and deep (green) zone of the subregions. The anterior and posterior subregions of both the medial and lateral compartments showed an almost identical COMP staining pattern, whereas in the central subregion of the lateral compartment, a re-expression of COMP could be detected in the upper zone. Representative COMP-stained sections of the different subregions (*n* = 12).

It has been shown earlier that COMP is degraded in early phases of OA but re-expressed in later stages. In order to analyze if there is a relationship between cartilage degeneration and the expression and localization of COMP in different subregions of the medial and lateral tibial compartments, immunohistochemical (IHC) staining was performed (Figure 2). In all subregions, COMP was found to be expressed throughout the whole cartilage thickness.

However, in the central subregion of both the medial and lateral tibial compartments, COMP staining was reduced or even absent in the interterritorial matrix of the deep zone and a more pericellular accumulation of COMP could be detected. In the upper zone, COMP staining was found in the territorial and interterritorial matrix with a reduced pericellular staining, whereas the central subregion of the lateral compartment showed a strong cellular staining, indicating a re-expression in the upper layer.

In the anterior and posterior subregions of both the medial and lateral compartments, an almost identical COMP staining pattern was observed in the deep zone with COMP being localized in the territorial and interterritorial matrix. Moreover, COMP staining was reduced or absent of COMP in the pericellular matrix. In the upper zone, COMP was localized throughout the ECM (Figure 2).

### 2.3. Localization of TSP-1

**Figure 3:**
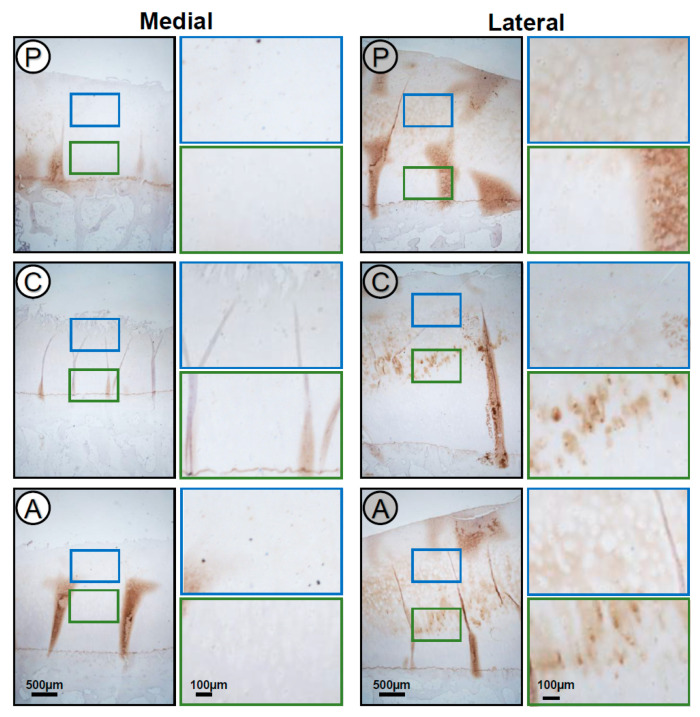
Localization of TSP-1 in the different articular cartilage subregions (A: anterior, C: central, P: posterior) of the medial and lateral tibial compartments. Magnifications of the upper (blue) and deep (green) zone of the subregions. TSP-1 expression was stronger in the cartilage ECM of all subregions from the lateral compartment. Representative TSP-1-stained sections of the different subregions (*n* = 12).

The IHC staining of TSP-1 showed that the protein is differentially expressed in the cartilage of the medial compared to the lateral compartment (Figure 3). TSP-1 staining was much stronger in the cartilage ECM in all subregions of the lateral compartment. Here, in the upper zone of all subregions, the TSP-1 staining was localized in the territorial and interterritorial matrix with a reduced or absent staining in the pericellular matrix. In the deep zone of the different subregions, an accumulation of TSP-1 was observed in the pericellular matrix. In some cases, an intracellular staining was detected. All subregions of the medial compartment showed only a very faint or no staining of TSP-1.

### 2.4. Localization of PLS3

**Figure 4:**
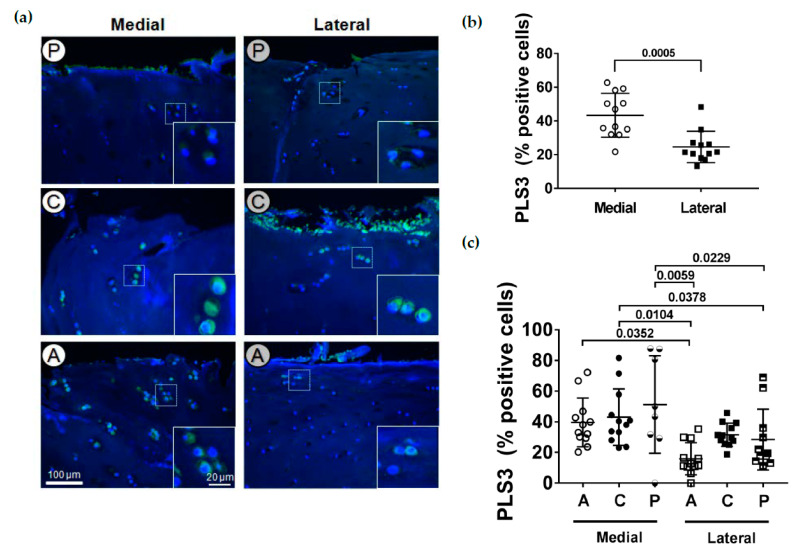
Immunofluorescence staining of PLS3 in chondrocytes. (**a**) PLS3-stained chondrocytes in the upper cartilage zone from the different cartilage subregions (A: anterior, C: central, P: posterior) of the medial and lateral tibial compartments. Magnifications in white boxes show PLS3 localization in individual chondrocytes. Representative PLS3-stained sections of the different subregions. (**b**) Quantitative evaluation of PLS3-positive stained cells in relation to the total number of cells. The total medial compartment had a significantly higher number of PLS3-positive chondrocytes compared to the lateral compartment (*n* = 12). (**c**) Percentage of PLS3-positive stained cells for the different subregions (A: anterior, C: central, P: posterior) at the medial and lateral tibial compartments (*n* = 8–12 per subregion). Values are represented as scatter plots with indicated mean. Significance between medial and lateral tibial compartments was evaluated using the paired t-test and between subregions using the ANOVA with repeated measurements and Duncan’s multiple range test for post-hoc analysis.

Even though the presence of PLS3 in chondrocytes has been described earlier [29], its exact localization in the tissue is not known. As an actin-binding and -bundling protein, one would expect a mechanical load-dependent expression. To identify the distribution of PLS3 in articular cartilage, immunofluorescence staining of PLS3 was performed on tissue sections. In all subregions of both the medial and lateral tibial compartments, the strongest staining of PLS3 was always detected in chondrocytes of the upper zone (Figure 4a). In the middle or deep zone, only a weak PLS3 staining in chondrocytes was found (Appendix A). However, when the PLS3-positive stained cells in relation to the total number of cells in the images of the upper, middle and deep zones from the different subregions were counted, the total medial compartment had a significantly (*p* = 0.0005) higher percentage of PLS3 positive chondrocytes compared to the lateral compartment (Figure 4b). When the different subregions of the medial and lateral compartments were compared, a higher percentage of positive cells could be detected in all subregions of the medial compartment in contrast to both the anterior and posterior but not to the central subregion of the lateral compartment (Figure 4c).

### 2.5. Localization of STIM1

**Figure 5:**
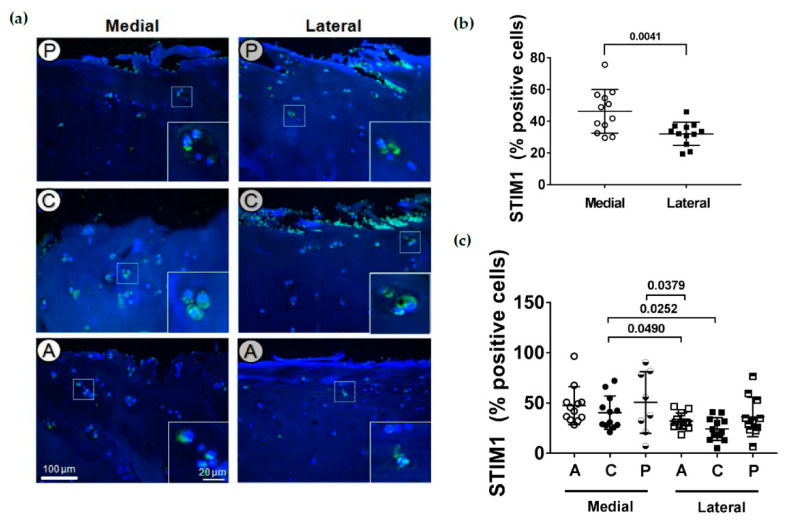
Immunofluorescence staining of STIM1 in chondrocytes. (**a**) STIM1-stained chondrocytes of the upper cartilage zone from the different cartilage subregions (A: anterior, C: central, P: posterior) of the medial and lateral tibial compartments. Magnifications in white boxes show STIM1 localization in individual chondrocytes. Representative STIM1-stained sections of the different subregions. (**b**) Quantitative evaluation of STIM1-positive stained cells in relation to the total number of cells. The total medial compartment had a significantly higher number of STIM1-positive chondrocytes compared to the lateral compartment (*n* = 12). (**c**) Percentage of STIM1-positive stained cells for the different subregions (A: anterior, C: central, P: posterior) at the medial and lateral tibial compartments (*n* = 8–12 per subregion). Values are represented as scatter plots with indicated mean. Significance between medial and lateral tibial compartments was evaluated using the paired t-test and between subregions using the ANOVA with repeated measurements and Duncan’s multiple range test for post-hoc analysis.

The transmembrane protein STIM1 is a Ca^2+^ sensor in the ER. It has been shown that STIM1 directly interacts with TSP-1 and COMP and seems to be involved in chondrogenesis. The TSPs influence the cellular calcium signaling through an interaction with STIM1 in the ER and the plasma membrane [29]. To analyze if STIM1 expression is altered during cartilage degeneration, immunofluorescence staining of STIM1 was performed on tissue sections. Like PLS3, in all subregions of both the medial and lateral tibial compartments, STIM1 was almost exclusively detected in chondrocytes of the upper zone (Figure 5a). In the middle or deep zones, only a weak STIM1 staining in chondrocytes could be found (Appendix B). However, when the STIM1-positive stained cells in relation to the total number of cells of the upper, middle and deep zones from the different subregions were counted, the total medial compartment had a significantly (*p* = 0.0041) higher percentage of STIM1-positive chondrocytes compared to the lateral compartment (Figure 5b). Regarding the different subregions of the medial and lateral compartments, a higher percentage of STIM1-positive cells could be detected in the central medial compartment compared to both the anterior and central but not to the anterior subregion of the lateral compartment (Figure 5c). In addition, the posterior subregion of the medial compartment had a higher percentage of STIM1-positive cells compared to the anterior subregion of the lateral compartment.

### 2.6. PLS3 and STIM1 Staining Correlates with the OARSI Score

**Figure 6:**
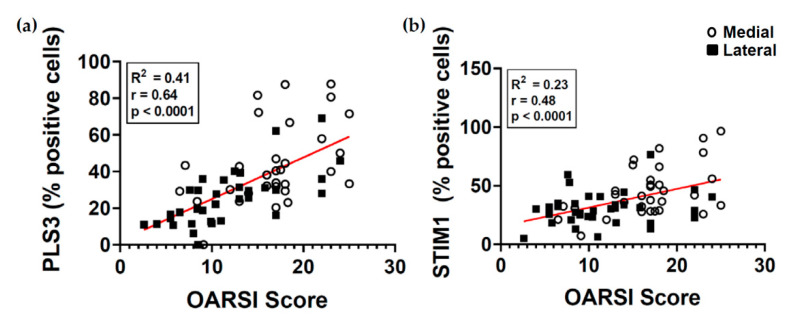
Correlations between the staining of PLS3 and STIM1 with the OARSI score. Scatter plot and linear regression of correlation between (**a**) PLS3 staining (% positive cells) and OARSI score and (**b**) STIM1 staining (% positive cells) and OARSI score. To test the correlation between variables, the Pearson’s correlation coefficient was calculated.

We found that the number of stained PLS3- and STIM1-positive cells was increased in the more affected medial compartment. We therefore analyzed if there is a correlation between the percentage of positive cells and the OARSI score. The OARSI score was strongly significant and positively correlated (r = 0.64, *p* ≤ 0.0001) with the PLS3 staining (Figure 6a). In addition, it was moderately significant and positively correlated (r = 0.48, *p* < 0.0001) with the STIM1 staining (Figure 6b). Interestingly, these data indicate a complex association between the expression of these proteins and the severity of OA again indicating a loading-dependent relationship.

### 2.7. Expression of TSPs, PLS3 and STIM1 in the Medial and Lateral Compartment

**Figure 7:**
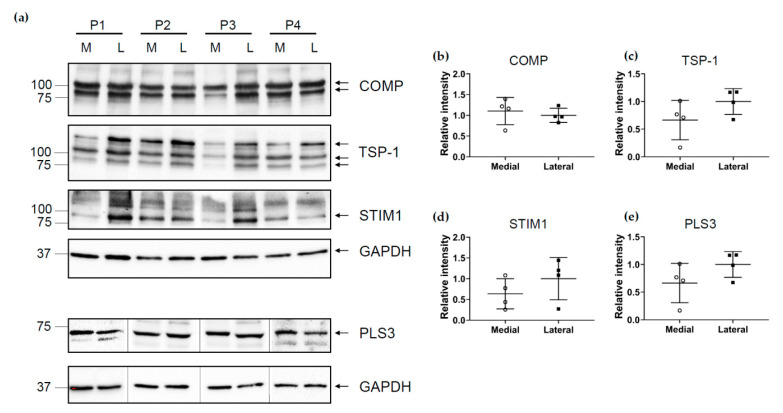
Immunoblots of cartilage extracts from the medial and lateral tibial compartments (M = medial, L = lateral) of four patients (P1–4: patient 1–4). (**a**) Blots were developed with specific antibodies directed against COMP, TSP-1, STIM1 and PLS3. Densitometric quantification of the bands was performed using ImageJ. The mean value of the band intensity in the lateral sample was set as 1 and all other intensities were related to this value. Band intensities for all proteins analyzed were normalized to intensities of GAPDH bands in the same samples run on the same gel. Relative intensities are shown for (**b**) COMP (**c**) TSP-1, (**d**) STIM1 and (**e**) PLS3. Significance between medial and lateral tibial plateaus was evaluated using the nonparametric Wilcoxon test.

To investigate the tissue levels of COMP, TSP-1, PLS3 and STIM1, total protein extracts were generated and analyzed using immunoblots. In many cases, the amount of remaining cartilage tissue was very low; in particular, in the central subregions of the medial side where the degeneration of the tissue was far advanced. Therefore, the tissue from the different subregions A, C and P was pooled and the protein levels were only compared between the medial and lateral compartments (Figure 7a). The total amount of COMP added from both compartments was variable in individual patients. This indicates a different degree of cartilage ECM degradation in different subjects. Nevertheless, the amount of COMP in the medial and lateral compartments was comparable in most patients. In addition, the analysis revealed the initiation of a proteolytic fragmentation within the tissue resulting in an additional band at ~90 kDa below the size of the intact monomer with ~120 kDa. A similar proteolytic fragmentation was observed for TSP-1. Here, besides the intact monomer, two additional fragment bands were detected. These fragments were seen in all samples and there were no fragment-specific correlations. Interestingly, the results clearly show that there was generally much less total TSP-1 present in the medial compared to the lateral compartment. This observation is completely in agreement with the IHC results (Figure 3). Again, the total level of TSP-1 was variable in patients. At least for two patients (P1 and P3), the level of STIM1 was clearly lower in the medial compartment. However, the variability between different patients precluded these differences becoming statistically significant (Figure 7b–e). The total level of PLS3 was comparable between both the different patients as well as between different compartments. However, when extracting the proteins, we found that PLS3 was rather susceptible to degradation, and therefore, the blots had to be performed immediately after extraction. This was also the reason for not being able to run all samples on the same gel.

## 3. Discussion

Due to a bowed leg alignment in varus knee OA patients, the medial plateau of the knee in these patients is subjected to more mechanical loading [40] and consequently prone to an increased OA risk and tissue damage, while the lateral knee plateau is less affected [37,38,41,42]. We could confirm that the visual cartilage degeneration was more severe at the medial plateau, and consequently, the OARSI score was significantly higher compared to the lateral plateau. Furthermore, our results could also verify a location-dependent prevalence of OA. Both the central and the posterior subregions of the medial compartment showed a higher OARSI score compared to all subregions of the lateral compartment. Within the medial compartment, the anterior subregion was less affected but the OARSI score was still significantly higher as compared to the lateral compartment.

The present study mainly investigated the topographical localization and expression of two ECM components, COMP and TSP-1, as well as two intracellular proteins that either link the ECM to the actin cytoskeleton or are known to contribute to Ca^2+^-dependent processes relevant for tissue homeostasis, PLS3 and STIM-1.

As stated before, degeneration of the ECM is a hallmark of OA. The cartilage ECM is a three-dimensional network mainly composed of collagen II, the proteoglycan aggrecan and noncollagenous proteins and glycoproteins [2]. COMP is a glycoprotein responsible for the stability of the ECM as bridging molecules and plays an important role in the cell–ECM interactions, which are Ca^2+^ dependent [4]. COMP is mostly located in the territorial and interterritorial matrix, especially in the deep layers [43]. This is in agreement with our results in the anterior and posterior subregions. Contrary to this, our IHC staining results indicated that COMP was predominantly localized in the pericellular matrix in the deep zones of the central cartilage regions, while being territorially and interterritorially distributed in the upper zone for both compartments. This might show that the central part of the cartilage is exposed to higher loads with increasing COMP fragmentation in general, which was abundantly found in the superficial zone in other studies [44].

It has been shown previously that the expression of COMP is mechanosensitive [45], and the intracellular staining suggests that COMP might be re-expressed due to altered mechanical loading in OA cartilage. The fact that COMP expression is reactivated in late-stage OA has been described earlier [7] and was interpreted as a compensatory mechanism to prevent further ECM damage. Using total cartilage protein extracts, we were not able to distinguish between COMP in the ECM and peri- or intracellular COMP. There might be a shift between these localizations, but the total COMP amounts on immunoblots remain unchanged when comparing the medial and lateral compartments. Unfortunately, and due to ethical concerns, it was not possible to obtain intact cartilage from healthy individuals, which would be a better control to investigate if a substantial amount of COMP is already lost from the tissue. It has to be considered also that the lateral compartment is exposed to an inflammatory milieu, and even if mechanical loading is less important, proteolytic degeneration by matrix metalloproteinases (MMPs) might already have been initiated [4]. The lower COMP band is most likely a first fragment generated by MMPs. It is still under debate where COMP proteolysis is initiated. Using immunoblotting, we clearly detected COMP fragments in cartilage extracts, suggesting that degeneration takes place in the tissue and not only in synovial fluid. Notably, we observed that the total COMP amount was rather variable in different patients, and using the amount of COMP as an indicator, one could assume that OA had further progressed in patient 2 and 3. Indeed, in these patients, we also detected additional fragments (Supplementary Figure S1: Original blots), which were not further analyzed in the present study.

Interestingly, TSP-1 showed a clear distinction between the medial and the lateral tibial compartments. The staining of TSP-1 in the cartilage ECM was much stronger in all subregions of the lateral compartment. Here, in all subregions, the TSP-1 staining was localized in the territorial and interterritorial matrix with a reduced or absent staining in the pericellular matrix in the upper zone. In the deep zone of the different subregions, TSP-1 was accumulated in the pericellular matrix. In some cases, an intracellular staining was detected. All subregions of the medial compartment showed only a very faint or no staining of TSP-1. This is in line with previous studies. It has been reported that in healthy cartilage, TSP-1 is located in the middle and deep zones [11]. Further, TSP-1 is elevated in mild OA, while it is reduced in severe OA [11]. Regarding our staining results, the lateral side might reflect mild OA, while the medial compartment exhibits more severe OA. The results of the immunoblots are in full agreement with the staining results and confirm lower TSP-1 amounts in the medial and higher amounts in the lateral compartment, even though these clear differences on the blot did not reach significance after densitometrical evaluation. The immunoblots revealed that also TSP-1 underwent proteolytic degradation, and in addition to the full-length proteins, two fragments were detected. It has been described in the literature that the N-terminal heparin-binding domain can be cleaved by ADAMTS-1 (a disintegrin and metalloproteinase with thrombospondin motifs-1), resulting in a 160 kDa fragment that can be further cleaved into a 130 kDa fragment by other proteases [5].

TSP-1 influences the availability of angiogenic factors and has been shown to inhibit angiogenesis directly by blocking endothelial cell proliferation, migration and apoptosis [46]. It is suggested that TSP-1 prevents the vascular invasion and is associated with osteoblast ingrowth into the articular cartilage necessary for bone formation [47]. It might well be that the upregulation of TSP-1 is of physiological relevance and should prevent the invasion of vessels that happens in certain stages of OA progression. The prevalent location of TSP-1 in the pericellular matrix of deep zone chondrocytes might reflect a direct interaction of TSP-1 with cell surface receptors [47,48,49] that is necessary for the induction of a cellular response.

The localization of TSP-1, PLS3 and STIM1 is different between the medial and lateral compartments, and the number of both PLS3- and STIM1-positive stained chondrocytes were increased depending on the higher load in the medial compared to the less loaded lateral compartment.

The ECM is connected via cell surface receptors, including integrins, to the cytoskeleton, and mechanical stimuli can be transferred in both directions, from the outside into the cell and from inside the cell to the ECM [2]. It has been shown that this mechanotransduction seems to be disturbed in OA [50,51] and that calcium signaling plays an important role in this process [52,53]. To better understand the interaction between the actin cytoskeleton and ECM in the pathomechanism of OA, we studied the localization and protein levels of COMP, TSP-1, PLS3 and STIM1.

Previous studies have shown that the first reaction of chondrocytes to mechanical signals is an increase in intracellular Ca^2+^ [54,55]. Intracellular Ca^2+^ oscillations are not only associated with ECM alterations [54] but also with the rearrangement of the actin cytoskeleton controlled by PLS3 [16]. Interestingly, we also detected that STIM1 is mainly located at the upper zone of the OA cartilage of both the medial and lateral compartments. This might also indicate that there is a relationship between OA and calcium-dependent mechanotransduction. Beside the influx of extracellular calcium, intracellular storage of Ca^2+^ in the ER can be released to regulate cytosolic Ca^2+^ [54]. STIM1 is located at the ER membrane, and the STIM1/Orai1 cluster enables refilling of ER storage via store-operated channels [56]. In turn, calcium store depletion abolishes chondrocyte differentiation [54,56], which is characteristic for the initiation process for osteogenesis seen in OA [57]. Furthermore, elevation of intracellular Ca^2+^ is associated with OA [54]. Interestingly, we found more STIM1-positive cells in the medial compared to the lateral compartment, further strengthening the hypothesis that mechanical loading also affects STIM1 expression. The fact that STIM1 has been shown to directly interact with TSPs could be of relevance in calcium-dependent mechanotransduction. COMP has been shown to bind calcium and undergoes a conformational change upon calcium binding [58]. However, according to the available calcium concentrations in the ECM, COMP would most likely be completely saturated with calcium. However, it might well be that intracellular COMP that plays a crucial role in collagen secretion via ER and Golgi [6] encounters STIM1. This idea has to be followed up in future studies.

We could show for the first time in cartilage tissue sections that PLS3 is localized in chondrocytes of different zones. Our findings clearly show an increased number of PLS3-positive stained cells in the medial compartment compared to the lateral side. An upregulation of PLS3 has been described in OA chondrocytes [29]. However, in this study, the proteome of isolated chondrocytes from patients suffering from OA and from healthy donors was compared. The proteome data were validated with immunoblot analysis, confirming a two-fold upregulation at the protein level. Even though our study confirms a correlation of PLS3 staining with OA severity, the total PLS3 amount extracted from cartilage was not significantly changed. One could speculate that intracellular PLS3 is released from dying cells and deposited in the tissue (see Figure 4a). Immunoblots of cartilage protein extracts would detect both intracellular PLS3 and PLS3 extracted from these extracellular deposits. Another explanation could be that Tsolis et al. [29] analyzed isolated chondrocytes, and in particular, that chondrocytes from healthy cartilage served as the control.

The function of PLS3 in cartilage is not yet well established. However, in bone diseases such as osteoporosis or osteogenesis imperfecta, PLS3 seems to play an important role [19]. *Pls3* KO mice demonstrated an osteoporotic phenotype, while *PLS3* overexpressing mice showed hyperostosis [26]. PLS3 was detected in the dendrites of osteocytes [59], and it has been suggested that PLS3 might be involved in the mechanotransduction [18,27,28,60] and seems also to be involved in osteoclastogenesis [26]. Interestingly, we found that PLS3 was mainly located at the upper zone of the OA cartilage. However, the upper layer in degenerating OA cartilage does not necessarily represent the superficial zone of a healthy articular cartilage. We therefore do not suggest a location-specific PLS3 expression in the superficial zone. We rather assume that PLS3 is involved in the mechanotransduction process due to its permanent localization at the force-facing cartilage side. This might strengthen its role in the mechanotranduction process for which proper actin dynamics regulated by actin bundling proteins such as PLS3 is crucial [61,62]. Schwebach et al. [16] have shown that mutations in *PLS3* affect Ca^2+^-regulated actin-bundling. In many cases, including PLS3, mutations lead to reduced protein amounts [63]. It is attractive to speculate that a mechanoresponse of chondrocytes could be regulated by PLS3 amounts.

Our study has several limitations. First, we analyzed only cartilage samples from patients suffering from OA and were not able to include healthy cartilage as a control. In addition, we did not collect blood samples from the patients and can therefore not correlate histological findings with serum levels of distinct proteins. Furthermore, the use of milder extraction buffers that we applied in the present study enhanced the risk of not quantitatively extracting all proteins, in particular if they have low solubility. We used this compromise to extract both intracellular proteins such as PLS3 and STIM1 equally well as oligomeric matrix components such as COMP and TSP-1. However, an incomplete extraction of certain proteins might explain the discrepancy between the results seen in the immunostainings and the immunoblots. In addition, the sample size with *n* = 4 for the immunoblot analysis was rather small. This could be an explanation for the missing statistical significance in the analyzed parameters. Further, all subregions of one compartment had to be pooled to obtain sufficient protein amounts for a detection with antibodies. A quantification of the protein per subregion was therefore not possible.

In conclusion, our study provides novel information on alterations in the ECM of OA cartilage suggesting that a Ca^2+^-dependent mechanotransduction between the ECM and the actin cytoskeleton via PLS3 and STIM1 might play an essential role in the pathomechanism of OA.

## 4. Materials and Methods

### 4.1. Tissue Samples

Tibial plateaus were collected from 16 patients (8 males, 8 females; age 67 ± 9 (range 48–81) years) suffering from primary OA at the medial knee compartment (varus knee OA) and undergoing total knee replacement surgery at the Department for Orthopaedic and Trauma Surgery at the University Hospital of Cologne. The study was approved by the local ethic committee (application number 14-422), and informed written consent was obtained from tissue donors. Only whole tibia plateaus, containing both the medial and lateral plateaus, without visible surgery artifacts affecting articular cartilage, were included in the study.

From the tibia plateaus, three osteochondral plugs (Ø 1 cm) were harvested at both the medial and lateral compartments at specific defined subregions—anterior (A), central (C) and posterior (P)—using a bone graft harvester (Arthrex^®^ AR-1981-10H, Arthrex GmbH, München, Germany) (Figure 1a), resulting in 96 plugs. Two plugs had to be excluded from the study because there was only a thin layer of subchondral bone that broke during harvesting. The plugs of 12 tibial plateaus (*n* = 70) were used for histological and immunohistological analysis. Samples were fixed in 4% paraformaldehyde for 72 h, decalcified with 0.5 M ethylenediaminetetraacetic acid (EDTA) at 37 °C for 8 weeks, embedded in paraffin and cut into 7-µm-thick sections. The six plugs from the remaining four tibial plateaus (*n* = 24) were used for protein extraction followed by immunoblotting.

### 4.2. Histological Scoring

To determine cartilage degeneration at the different subregions, the OARSI score was applied to evaluate the sections of the 70 plugs. Scoring was performed by two independent observers on Safranin O/Fast green-stained sections that were made according to standard histochemical protocols. The score (0–24) is the product of the OA severity (six grades) and the horizontal extent of the involved cartilage surface (four stages) [39].

### 4.3. Antibodies

The following primary antibodies were used: monoclonal mouse antibodies directed against GAPDH (MA5-15738; ThermoFisher Scientific, Rockford, IL, USA) and against human TSP-1 (BA-24, Merck KGaA, Darmstadt, Germany), and polyclonal rabbit antibodies directed against rat and bovine COMP [64], against human STIM1 (11565-1-AP, Proteintech Germany GMBH, St. Leon-Rot, Germany) and against human PLS3 [27].

### 4.4. IHC and Immunofluorescence Analysis

After deparaffination, the sections were rehydrated prior to antigen retrieval. The sections were incubated with 0.025% pepsin in 0.2 N HCl at 37 °C for 15 min, followed by digestion with hyaluronidase (500 U/mL, Sigma-Aldrich, St. Louis, MO, USA) in 100 mM NaH_2_PO_4_, 100 mM NaCH_3_COO (pH = 5) at 37 °C for 30 min and proteinase K (10 µg/mL, Qiagen GmbH, Hilden Germany) in 50 mM Tris/HCl, 1 mM EDTA (pH = 7.4) at 55 °C for 10 min. Nonspecific binding of the antibodies was blocked by incubation with blocking solution (ZUC007-100, Zytomed Systems, Berlin, Germany) for 5 min. Then, the sections were incubated with primary antibodies overnight at 4 °C. The antibodies were diluted 1:200 (TSP-1; STIM1), 1:250 (PLS3) and 1:1000 (COMP) in TBS containing 1% bovine serum albumin (BSA). Primary TSP-1 and COMP antibodies were detected using the ZytoChem Plus horseradish peroxidase polymer anti-mouse (ZUC050-006, Zytomed Systems, Berlin, Germany) and anti-rabbit (ZUC032-006, Zytomed Systems, Berlin, Germany), respectively. HRP-polymer coupled secondary antibodies were detected using 3,3′-diaminobenzidine (DAB). Sections were counterstained with hematoxylin, dehydrated and mounted with DPX (TSP-1 and COMP). For immunofluorescence staining (STIM1 and PLS3), AlexaFluor488 fluorescent-labelled goat ant-rabbit IgGs (A-11034, Thermo Fisher Scientific, Rockford, IL, USA) coupled secondary antibody was applied. Nuclei were stained using 4′,6-diamidino-2-phenylindole (DAPI). Sections were mounted with fluorescence mounting medium (Dako AG, Wiesentheid, Germany).

The immunofluorescence stainings of PLS3 and STIM1 were quantitatively evaluated. In one section of each location-dependent plug, three images were taken, whereby at least one image was located in the upper, middle, and deep zones of the cartilage. Three plugs (all posterior (P)) from three patients could not be evaluated because there was no more cartilage on the section after the immunofluorescence staining. PLS3- and STIM1-positive chondrocytes related to the total number of DAPI stained cells (% positive cells), which were counted in each image and the mean value for these three images was noted. Images were taken with a 200-fold magnification using a microscope (Axiphot2, Carl Zeiss, Jena, Germany) with the software NIS-Elements (Nikon, Düsseldorf, Germany). Positive cells were counted using the ImageJ software (version 1.53, https://imagej.nih.gov/ij/index.html, accessed on 1 February 2021) [65].

### 4.5. Immunoblotting

Proteins were extracted from the cartilage of the plugs using RIPA lysis buffer containing 1% (*v/v*) NP-40, 0.05% (*v/v*) Triton C-100, 0.5% (*w/v*) SDS, 2.5 mM EDTA and 150 mM NaCl in 20 mM Tris-HCl (pH 7.4). For immunoblotting, the extracts of the subregions A, C and P of both the medial and lateral compartments had to be pooled to obtain sufficient protein amounts. Protein concentrations were determined using a bicinchoninic acid-based protein quantification kit (Interchim, Monluçon CEDEX, France). Equal protein amounts were separated by SDS-PAGE and transferred onto nitrocellulose membranes. Membranes were blocked with 5% BSA in TBS. Antibodies against GAPDH (1:1000), TSP-1 (1:1000), STIM1 (1:1000), PLS3 (1:1000) and COMP (1:1000) were used as primary antibodies. Primary antibodies were detected using the ZytoChem Plus horseradish peroxidase polymer anti-mouse (ZUC050-006, Zytomed Systems, Berlin, Germany) and anti-rabbit (ZUC032-006, Zytomed Systems, Berlin, Germany), respectively. Detection of the bound antibodies was achieved by enhanced chemiluminescence. Signals were analyzed with the Chemi DocTM XRS+ (Bio-Rad). Molecular imager and the ImageLabTM software (http://www.bio-rad.com/de-de/product/Image-lab-software, accessed on 1 February 2021) and band intensities quantified with the ImageJ software (version 1.53, https://imagej.nih.gov/ij/index.html, accessed on 1 February 2021) [65]. Band intensities for all proteins analyzed were normalized to intensities of GAPDH bands in the same samples run on the same gel. The average intensity of all bands analyzed from the lateral side was set as 1.

### 4.6. Statistical Analysis

Statistical analysis was performed using Statistica 7.1 (StatSoft (Europe) GmbH, Hamburg, Germany) and GraphPad Prism 7.04 (GraphPad Software, San Diego, CA, USA). Normal distribution of the data was tested with the Kolmogorov–Schmirnov test, and sphericity was checked using Mauchly´s sphericity test. A one-way analysis of variance (ANOVA) with repeated measurements and Duncan’s multiple range test for post-hoc analysis was performed to detect significant differences between the six subregions. To detect significant differences between the medial and lateral tibial plateaus, depending on the sample size, either a Student’s paired *t*-test or a nonparametric Wilcoxon signed-rank test was applied. To test the correlation between variables, the Pearson’s correlation coefficient was calculated. Data are presented as mean ± SD. The level of significance was selected at *p* ≤ 0.05.

## Figures and Tables

**Figure A1:**
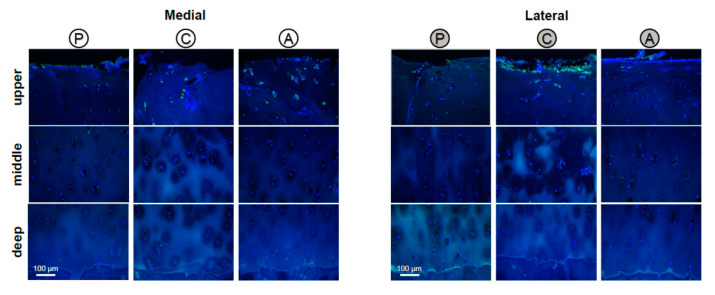
Immunofluorescence staining of PLS3 in chondrocytes on articular cartilage sections. PLS3 could be detected in chondrocytes of the upper, middle and deep cartilage zones from the different cartilage subregions (A: anterior, C: central, P: posterior) of the medial and lateral tibial compartments. The strongest staining was found in chondrocytes of the upper zone. Representative PLS3-stained sections of the different subregions from one patient are shown.

**Figure A2:**
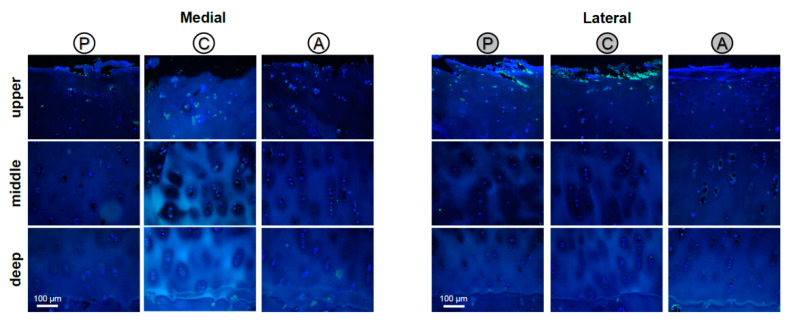
Immunofluorescence staining of STIM1 in chondrocytes on articular cartilage sections. STIM1 could be detected in chondrocytes of the upper, middle and deep cartilage zones from the different cartilage subregions (A: anterior, C: central, P: posterior) of the medial and lateral tibial compartments. The strongest staining was found in chondrocytes of the upper zone. Representative STIM1-stained sections of the different subregions from one patient are shown.

## Data Availability

The data that support the findings of this study are available from the corresponding author upon reasonable request.

## Supplementary Materials

Supplementary materials can be found at https://www.mdpi.com/1422-0067/22/6/3073/s1.

## Abbreviations

ABD	Actin-binding domain
ADAMTS	A disintegrin and metalloproteinase with thrombospondin motif
BSA	Bovine serum albumin
COMP	Cartilage oligomeric matrix protein
DAB	3,3′-diaminobenzidine
DAPI	4′,6-diamidino-2-phenylindole
DPX	Dibutylphthalate polystyrene xylene
ECM	Extracellular matrix
EDTA	Ethylenediaminetetraacetic acid
ER	Endoplasmic reticulum
GAPDH	Glycerinaldehyde-3-phosphate-dehydrogenase
HCL	Hydrogen chloride
HRP	Horseradish peroxidase
IgG	Immunoglobulin G
IHC	Immunohistochemistry
MMP	Matrix metalloproteinase
NaCH_3_COO	Sodium acetate
NaH_2_PO_4_	Monosodium phosphate
OA	Osteoarthritis
OARSI	Osteoarthritis Research Society International
PBS	Phosphate-buffered saline
PLS	Plastin
SDS-PAGE	Sodium dodecyl sulfate polyacrylamide gel electrophoresis
STIM1	Stromal interaction molecule 1
TBS	Tris-buffered saline
Tris/HCL	Tris(hydroxymethyl)aminomethane hydrochloride
TSP	Thrombospondin

## Appendix A

### Localization of PLS3 in Human Articular Cartilage

To detect PLS3 in different human articular cartilage compartments of an osteoarthritic varus knee, immunofluorescence staining of PLS3 in tissue sections was performed. In all subregions of both the medial and lateral tibial compartments, the strongest expression of PLS3 was always detected in chondrocytes of the upper zone (Figure A1). In the middle or deep zones, only a weak PLS3 staining in chondrocytes was found, indicating that the PLS3 expression might depend on mechanical loading.

## Appendix B

### Localization of STIM1 in Human Articular Cartilage

To analyze STIM1 expression in human articular cartilage, immunofluorescence staining of STIM1 was performed on tissue sections. Like PLS3, STIM1 could be detected almost exclusively in chondrocytes of the upper zone and in all subregions of both the medial and lateral tibial compartments (Figure A2). In the middle or deep zones, only a weak STIM1 staining in chondrocytes was observed. We expected the strongest STIM1 expression at locations with high mechanical loading because STIM1 is a Ca^2+^ sensor in the ER and the regulation of the intracellular calcium concentration is crucial for cartilage mechanotransduction [55].
