# Supplementary figures and images for: Expression and Localization of Thrombospondins, Plastin 3, and STIM1 in Different Cartilage Compartments of the Osteoarthritic Varus Knee
Source: Int J Mol Sci. 2021 Mar 17;22(6):3073. doi: 10.3390/ijms22063073 (PMC8002632; doi:10.3390/ijms22063073)

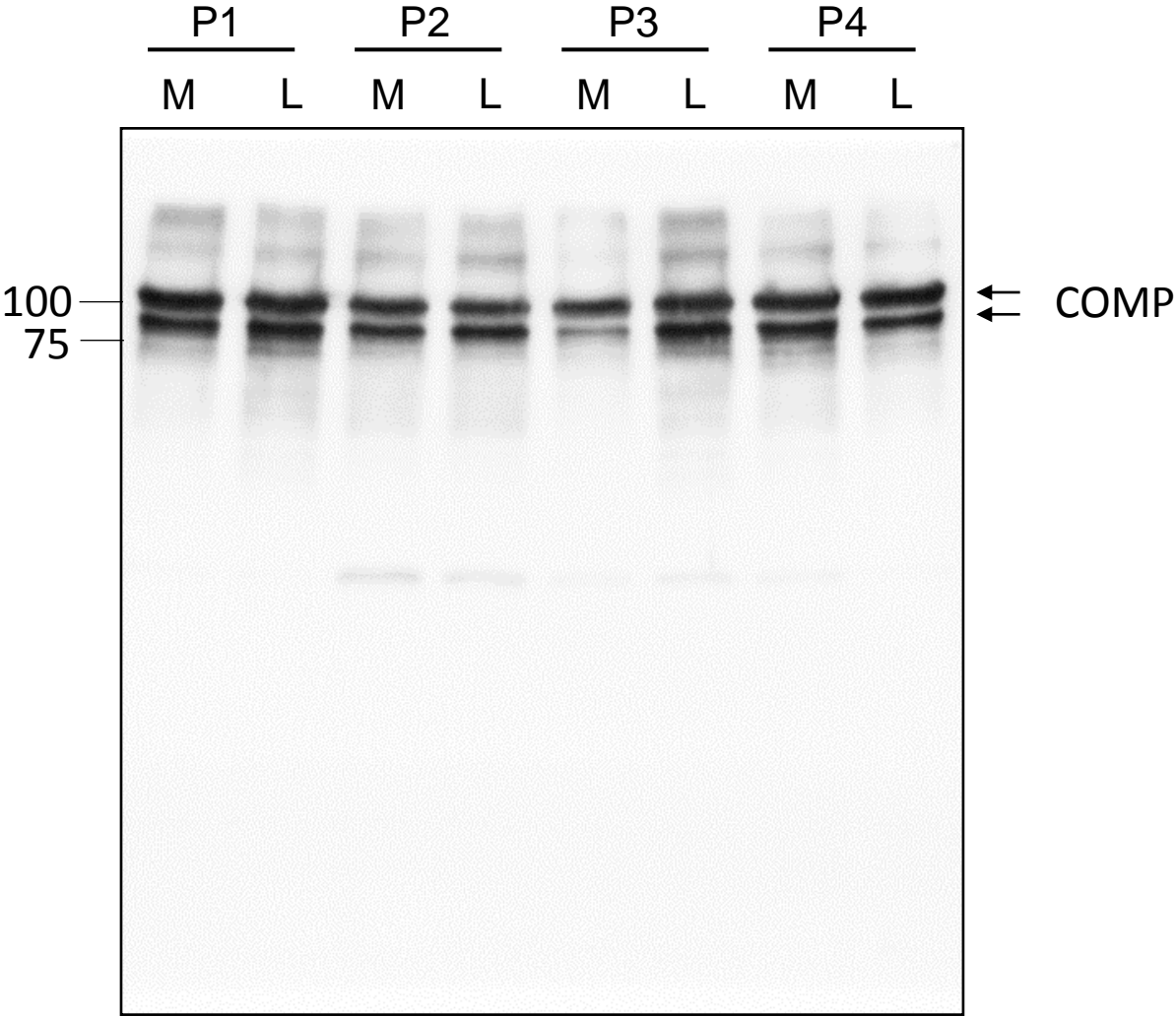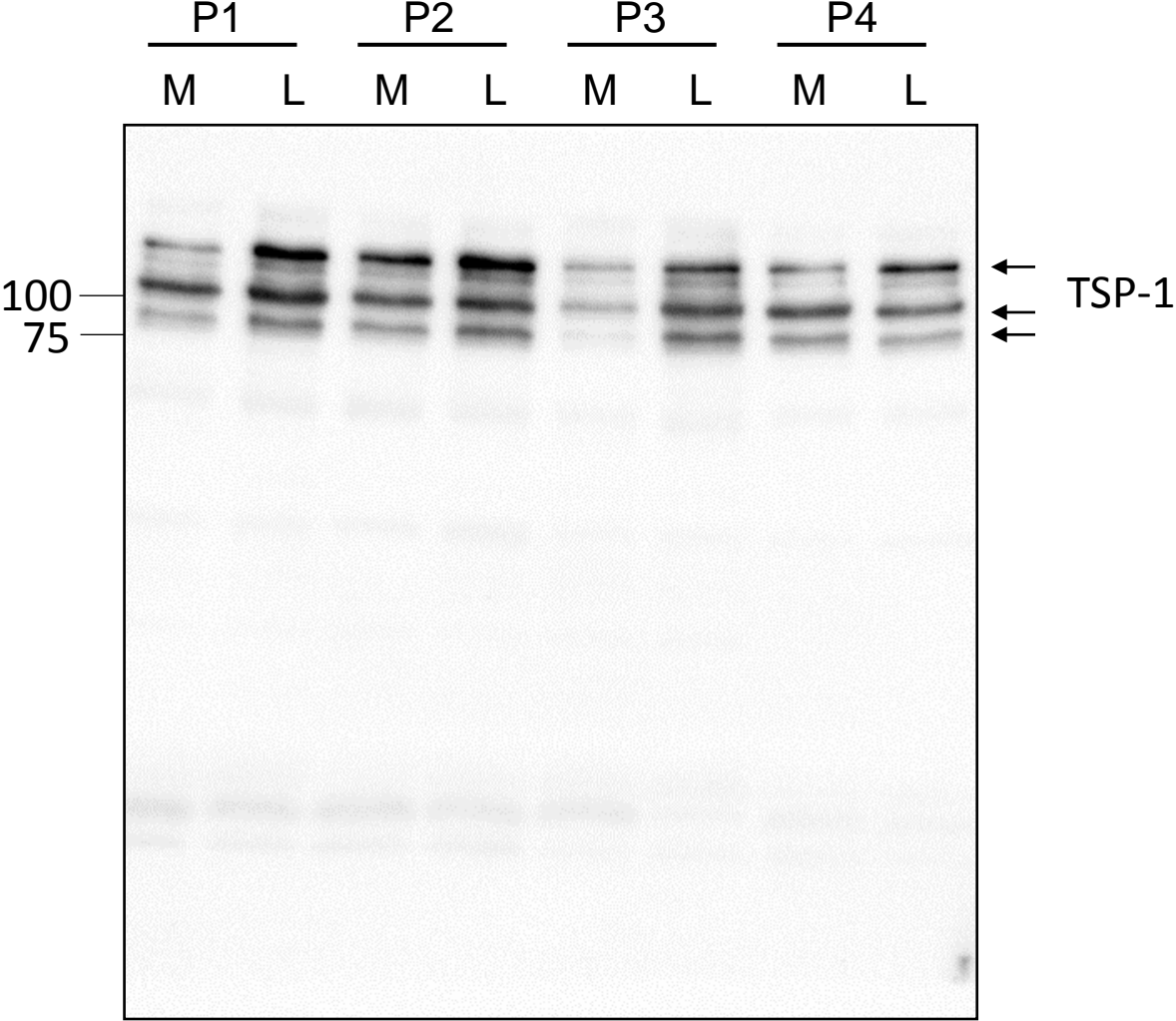

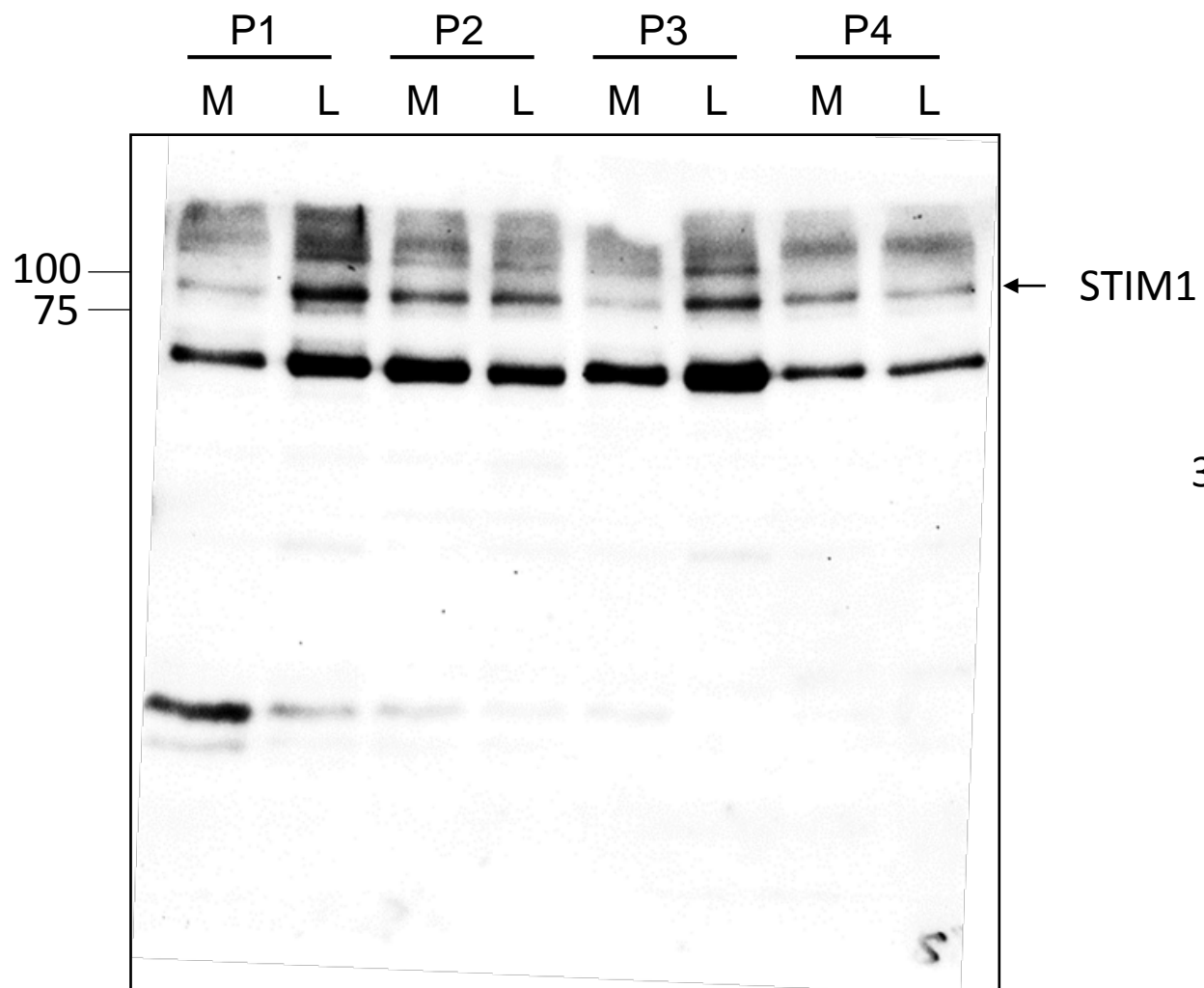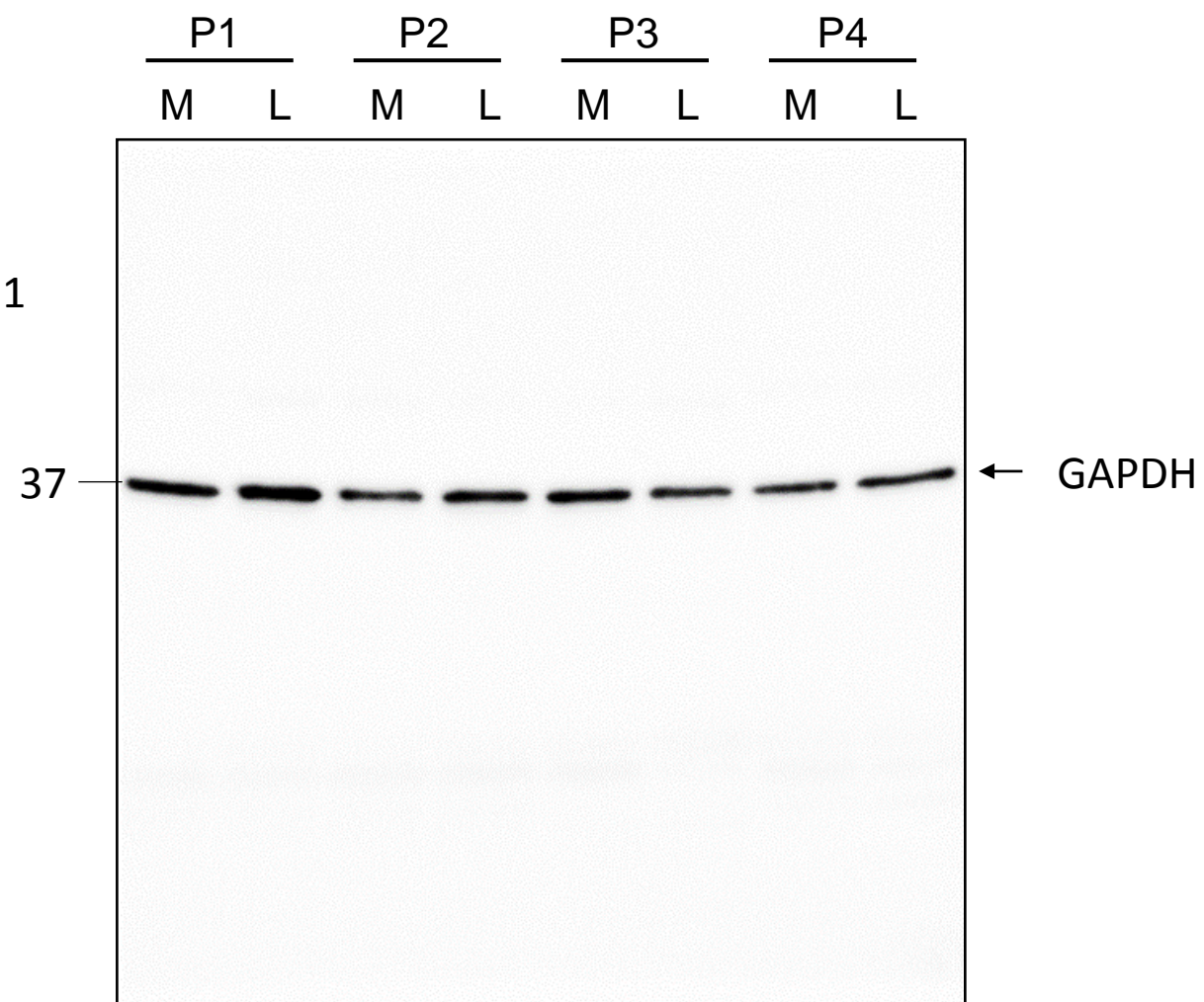

# PLS3

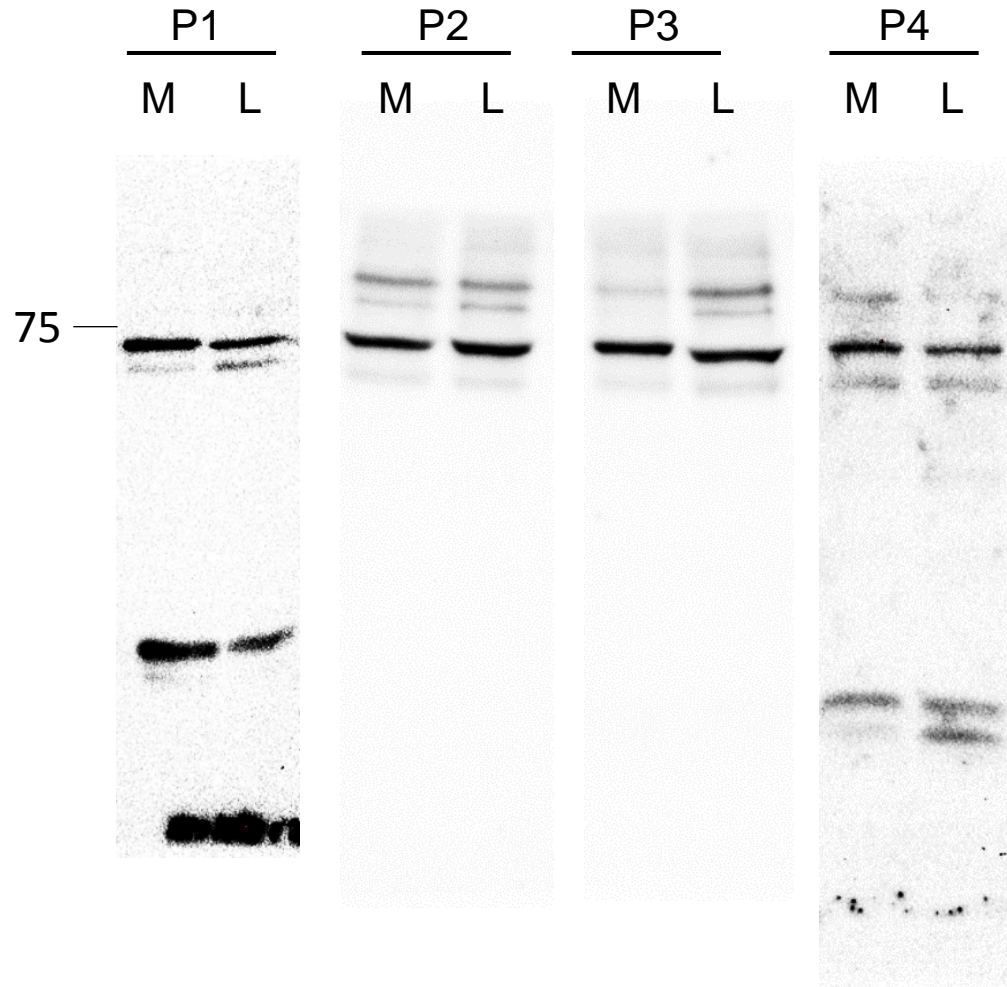

# GAPDH

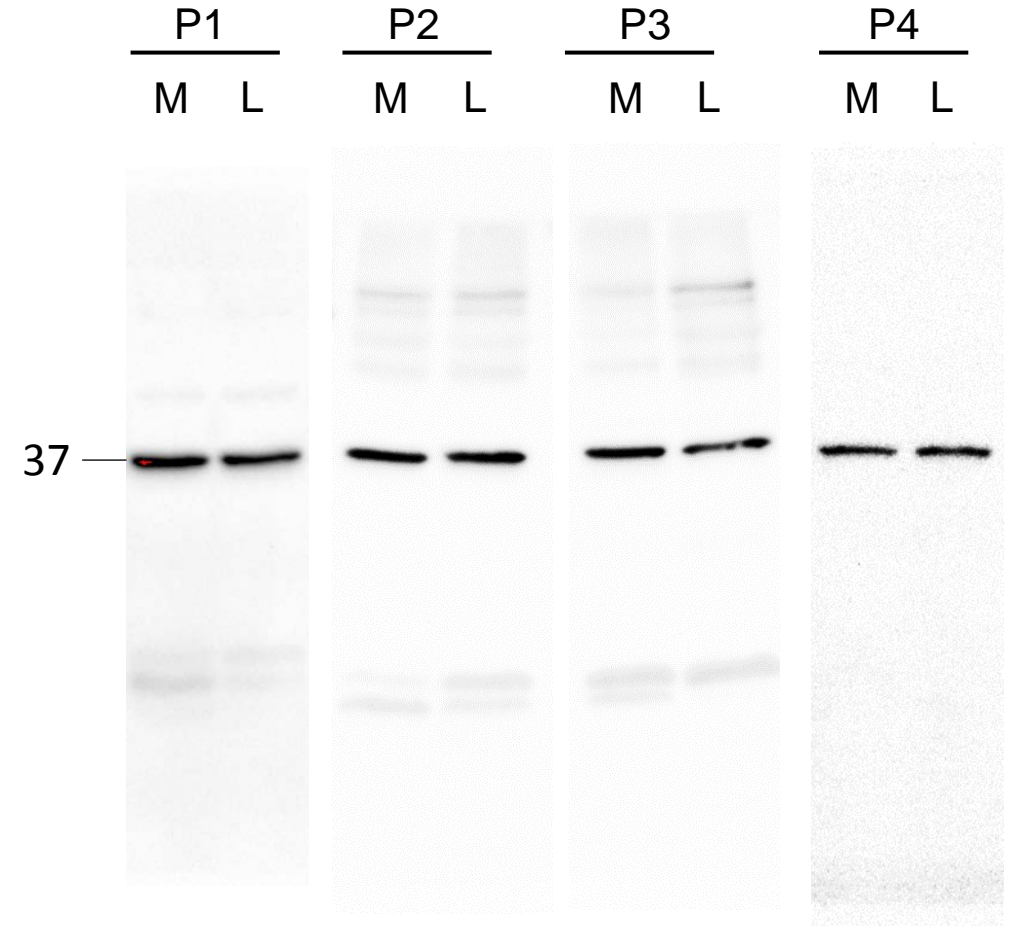

Supplement: Supplementary file 1 [file ijms-22-03073-s001.zip › Supplementary material/Blots original 160321.pdf]
