# Supplementary material for: Expression and Localization of Thrombospondins, Plastin 3, and STIM1 in Different Cartilage Compartments of the Osteoarthritic Varus Knee
Source: Int J Mol Sci. 2021 Mar 17;22(6):3073. doi: 10.3390/ijms22063073 (PMC8002632; doi:10.3390/ijms22063073)
Supplement: Supplementary file 1 [file ijms-22-03073-s001.zip › Supplementary material/Legend Supplementary Figure S1.doc]

Supplementary Figure S1:

Original full-size immunoblots of cartilage extracts from the medial and lateral tibial compartments (M = medial, L = lateral) of four patients (P1–4: patient 1–4).

As described in Figure 7 of the main manuscript, the blots were developed with specific antibodies directed against COMP, TSP-1, STIM1 and PLS3. Band intensities for all proteins analyzed were normalized to intensities of GAPDH bands in the same samples run on the same gel.
